# Stress Response of Mouse Embryonic Fibroblasts Exposed to Polystyrene Nanoplastics

**DOI:** 10.3390/ijms22042094

**Published:** 2021-02-20

**Authors:** Seung-Woo Han, Jinhee Choi, Kwon-Yul Ryu

**Affiliations:** 1 Department of Life Science, University of Seoul, Seoul 02504, Korea; eipot@naver.com; 2 School of Environmental Engineering, University of Seoul, Seoul 02504, Korea; jinhchoi@uos.ac.kr

**Keywords:** nanoplastic, polystyrene, MEFs, endocytosis, oxidative stress, autophagy

## Abstract

Polystyrene (PS) nanoplastic exposure has been shown to affect the viability of neuronal cells isolated from mouse embryonic brains. However, the viability of mouse embryonic fibroblasts (MEFs) was not affected although PS nanoplastics accumulated in the cytoplasm. It is currently unknown whether MEFs do not respond to PS nanoplastics or their cellular functions are altered without compromising viability. Here, we found that PS nanoplastics entered the cells via endocytosis and were then released into the cytoplasm, probably by endosomal escape, or otherwise remained in the endosome. Oxidative and inflammatory stress caused by intracellular PS nanoplastics induced the antioxidant response pathway and activated the autophagic pathway. However, colocalization of the autophagic marker LC3B and PS nanoplastics suggested that PS nanoplastics in the cytoplasm might interfere with normal autophagic function. Furthermore, autophagic flux could be impaired, probably due to accumulation of PS nanoplastic-containing lysosomes or autolysosomes. Intriguingly, the level of accumulated PS nanoplastics decreased during prolonged culture when MEFs were no longer exposed to PS nanoplastics. These results indicate that accumulated PS nanoplastics are removed or exported out of the cells. Therefore, PS nanoplastics in the cytoplasm affect cellular functions, but it is temporal and MEFs can overcome the stress caused by PS nanoplastic exposure.

## 1. Introduction

Polystyrene (PS) is widely used in our daily life, but it can be an environmental concern as it is not biodegradable [1,2]. Ingestion or inhalation of PS nanoplastics may cause health problems, but more serious problems can be caused when they are taken up by cells [3]. In our previous study, we have demonstrated that PS nanoplastics with a diameter of 100 nm can enter cells, but the susceptibility is different depending on the cell type [4]. Although neuronal cells were vulnerable to the exposure of PS nanoplastics with increased apoptosis, mouse embryonic fibroblasts (MEFs) maintained cellular integrity with PS nanoplastics accumulating inside the cells. It is currently unknown why the presence of PS nanoplastics in the cytoplasm caused cell-type-specific toxicity. Furthermore, it was unclear whether MEFs did not respond to PS nanoplastics or their cellular functions were altered without affecting viability.

Nano-sized particles can enter and exit cells through endocytosis and exocytosis [5]. These properties can be useful for efficient drug delivery to the desired cell type or removal from unwanted cells. Similarly, PS nanoplastics are also expected to enter cells via endocytosis. When PS nanoplastics are in endosomes, they can be released into the cytoplasm by endosomal escape. PS nanoplastics in the cytoplasm can interfere with normal cellular function, putting cells under stress conditions such as oxidative or inflammatory stress [6]. Alternatively, PS nanoplastic-containing (PS-containing) endosomes can be fused with lysosomes, but it is unlikely that PS nanoplastics are degraded in the lysosomal compartment. Therefore, PS-containing late endosomes or PS-containing lysosomes may accumulate in cells.

For autophagic clearance of damaged organelles/macromolecules or misfolded protein aggregates, autophagosomes must be fused with lysosomes [7]. However, the accumulation of PS-containing lysosomes may interrupt this process and autophagic flux can be impaired. In fact, nanoparticles or PS nanoplastics have also been shown to affect the autophagic pathway. Nanoparticles can be used to deliver drugs to modulate autophagy and induce apoptosis, which could potentially be used in cancer treatment [8]. Delivery of PS nanoplastics to cells, using transfection reagents, has also been shown to activate autophagy [9]. Moreover, PS nanoplastics interfered with autophagic flux, which was ameliorated when PS nanoplastics were forced to form complexes with protein coats [10]. PS nanoplastics in the cytoplasm activated the transcription factor EB, known as the master regulator of lysosome biogenesis and autophagy [11]. It has been suggested that the positive charge on PS nanoparticles plays a role in causing lysosomal dysfunction and autophagic flux inhibition [11,12].

Although many studies have suggested a cellular dysfunction or stress response to PS nanoplastics in the cytoplasm, it is currently unknown how certain cell types can overcome the stress caused by PS nanoplastics. In this study, we used MEFs and aimed to elucidate how they were able to maintain cellular integrity in the presence of PS nanoplastics. To achieve this goal, we investigated the uptake mechanism of PS nanoplastics into the cytoplasm and monitored their behavior inside the cells.

## 2. Results

### 2.1. Uptake of PS Nanoplastics into MEFs via Endocytosis

**Figure 1:**
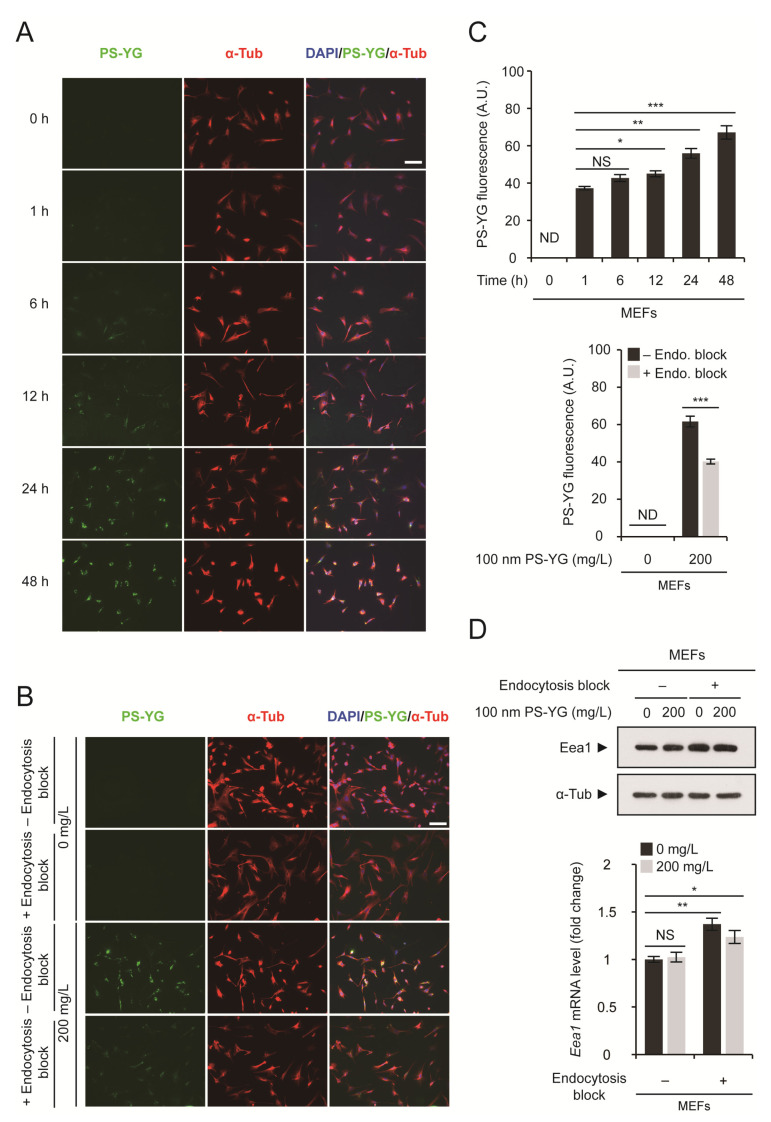
Polystyrene (PS) nanoplastic uptake via endocytosis. (**A**) Mouse embryonic fibroblasts (MEFs) were treated with 100 nm PS-YG (yellow-green) nanoplastics at 200 mg/L for 0 to 48 h. Cells were subjected to immunofluorescence analysis using an anti-α-tubulin (α-Tub) antibody and DNA was visualized with DAPI. Direct fluorescence from PS-YG nanoplastics was also visualized. (**B**) Endocytosis was blocked using sodium azide. MEFs were pretreated with sodium azide (+ Endocytosis block) or vehicle (− Endocytosis block) for 16 h before 100 nm PS-YG treatment. After 16 h, MEFs were treated with 100 nm PS-YG nanoplastics at the indicated concentration for 24 h. (**C**) Quantitative analysis of PS-YG fluorescence levels in (**A**, upper panel) and (**B**, lower panel). Green fluorescence levels in all α-Tub-positive cells in the field were determined and expressed as the means ± SEM from the number of cells shown. (**D**) Immunoblot detection (upper panel) and qRT-PCR analysis (lower panel) of *Eea1* in MEFs treated with PS-YG at the indicated concentration for 1 day with or without sodium azide pretreatment to block endocytosis. α-Tubulin (α-Tub) was used as a loading control for immunoblot analysis. mRNA expression levels of *Eea1* were determined by qRT-PCR (*n* = 3 each), normalized against *Gapdh* levels and expressed as the fold change relative to untreated control levels. Representative images of cells or immunoblots are shown. qRT-PCR data are expressed as the means ± SEM from the indicated number of samples. * *p* < 0.05; ** *p* < 0.01; *** *p* < 0.001 between two groups, as indicated by horizontal bars. ND, not determined. NS, not significant. Scale bar, 100 μm.

We have previously reported that PS nanoplastics accumulated in the cytoplasm of various cell types, including MEFs and neurons, when cells were exposed to 100 nm PS nanoplastics [4]. Intracellular accumulation of PS nanoplastics reduced the viability of neurons but did not change the viability of MEFs. To determine how MEFs can tolerate the presence of PS nanoplastics, we decided to monitor them inside the cells. For direct visualization of PS nanoplastics, we treated MEFs with 100 nm PS-YG, in which PS nanoplastics were linked to yellow-green (YG) quantum dots. We first carried out a time-course analysis of PS nanoplastics inside the cells (Figure 1A,C). PS nanoplastics accumulated in the cytoplasm over time and reached their maximum level 24 h after exposure. When MEFs were pretreated with sodium azide, which blocks endocytosis, we found that uptake of PS nanoplastics into the cytoplasm was markedly reduced (Figure 1B,C). ATP is required for endocytosis, whereas sodium azide inhibits intracellular ATP production, thereby blocking endocytosis [13]. Although we cannot exclude the possibility that PS nanoplastics can simply diffuse into cells across the plasma membrane, it is evident that most PS nanoplastics enter cells via endocytosis. Blockage of endocytosis by sodium azide resulted in the upregulation of *Eea1* expression, which is an early endosome marker, at both the mRNA and protein levels (Figure 1D). In fact, endosomal dysfunction is one of the early symptoms of neurodegenerative diseases, including Alzheimer’s disease (AD) [14,15]. In AD patients, *Eea1* expression has also been reported to be upregulated [16]. These results confirm that endosomal membrane trafficking is affected when endocytosis is blocked. However, exposure of PS nanoplastics or their presence in the cytoplasm did not affect the expression of *Eea1*.

### 2.2. Activation of the Stress Response System in MEFs Caused by the Intracellular Accumulation of PS Nanoplastics

**Figure 2:**
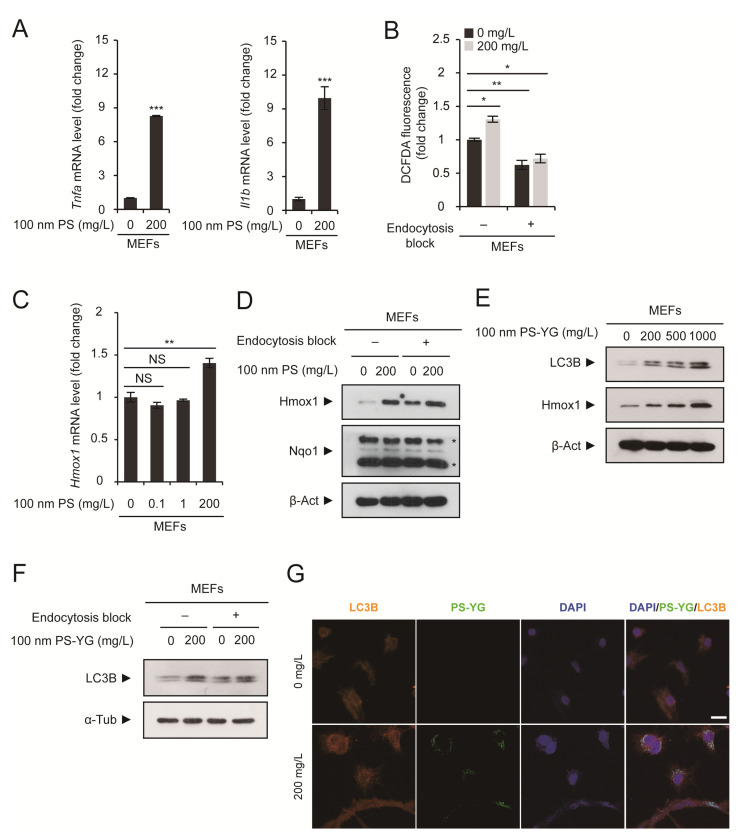
Stress response in MEFs caused by the intracellular accumulation of PS nanoplastics. (**A**,**C**) MEFs were treated with 100 nm PS nanoplastics at the indicated concentration for 2 days. mRNA expression levels of tumor necrosis factor-α (*Tnfa*), interleukin-1β (*Il1b*), and heme oxygenase1 (*Hmox1*) were determined by qRT-PCR (*n* = 3 each), normalized against *Gapdh* levels, and expressed as the fold change relative to untreated control levels. (**B**) MEFs were treated with PS nanoplastics at the indicated concentration for 1 day with or without sodium azide pretreatment to block endocytosis. ROS generation was detected using dichlorofluorescein diacetate (DCFDA)-based ROS detection assay kit. (**D**,**F**) Immunoblot detection of Hmox1, Nqo1, and LC3B in MEFs treated with PS or PS-YG at the indicated concentration for 1 day with or without sodium azide pretreatment to block endocytosis. β-Actin (β-Act) or α-tubulin (α-Tub) was used as a loading control. Asterisks (*) in Nqo1 immunoblot indicate non-specific bands. (**E**) Immunoblot detection of LC3B and Hmox1 in MEFs treated with PS-YG at the indicated concentration for 2 days. β-Actin (β-Act) was used as a loading control. (**G**) MEFs were treated with 100 nm PS-YG nanoplastics at the indicated concentration for 2 days. Cells were subjected to immunofluorescence analysis using an anti-LC3B antibody and DNA was visualized with DAPI. Direct fluorescence from PS-YG nanoplastics was also visualized. Representative immunoblots or images of cells are shown. qRT-PCR and ROS detection data are expressed as the means ± SEM from the indicated number of samples. * *p* < 0.05; ** *p* < 0.01; *** *p* < 0.001 vs. control (0 mg/L) or between two groups, as indicated by horizontal bars. NS, not significant. Scale bar, 50 μm.

When PS nanoplastics enter the cells via endocytosis, they may remain in the endosome. However, they can also be released into the cytoplasm by endosomal escape. Accumulation of PS nanoplastics in the cytoplasm upregulated the expression of proinflammatory cytokines such as Tnf-α and Il-1β (Figure 2A). Furthermore, they also generated reactive oxygen species (ROS), which was determined by cellular ROS detection assay (Figure 2B). ROS generation was most obvious when MEFs were exposed to PS nanoplastics and they entered the cells. Oxidative stress induced the antioxidant response pathway, as evidenced by the upregulation of antioxidant genes such as *Hmox1* and *Nqo1* (Figure 2C–E). Interestingly, when endocytosis was blocked, the induction of antioxidant response pathway due to exposure to PS nanoplastics was alleviated (Figure 2D).

PS nanoplastic accumulation also appeared to activate the autophagic pathway. The level of the autophagic marker LC3B increased in a PS concentration-dependent manner, although the conversion from LC3B-I to LC3B-II was not evident (Figure 2E). This PS-induced increase in LC3B levels was most obvious when endocytosis was allowed (Figure 2F). Most strikingly, we found that LC3B-positive puncta colocalized with PS nanoplastics, suggesting that autophagosomes may contain PS nanoplastics (Figure 2G). The fusion of the autophagosome and the functional lysosome can result in the formation of autolysosome, but the autophagic flux may be impaired as PS nanoplastics cannot be cleared even under acidic environment. In addition, lysosomes may be dysfunctional if they already contain PS nanoplastics.

**Figure 3:**
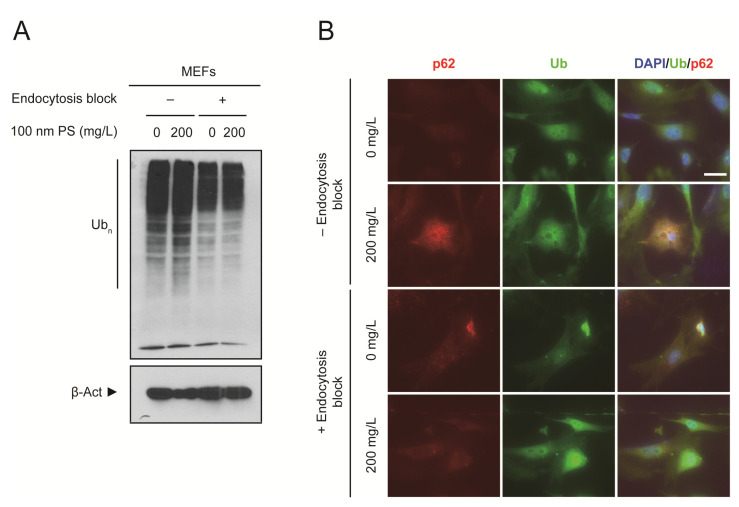
Interference with normal autophagic function in MEFs in the presence of PS nanoplastics. (**A**) Immunoblot detection of ubiquitin conjugates (Ub_n_) in MEFs treated with PS nanoplastics at the indicated concentration for 1 day with or without sodium azide pretreatment to block endocytosis. β-Actin (β-Act) was used as a loading control. (**B**) MEFs were treated with 100 nm PS nanoplastics at the indicated concentration for 1 day with or without sodium azide pretreatment to block endocytosis. Cells were subjected to immunofluorescence analysis using anti-p62 and anti-Ub (FK2) antibodies, and DNA was visualized with DAPI. Representative immunoblots or images of cells are shown. Scale bar, 50 μm.

We also confirmed that the endocytosis-dependent induction of oxidative stress was caused by the exposure to PS nanoplastics as we observed increased levels of the ubiquitin (Ub) conjugates (Figure 3A). Under oxidative stress conditions, misfolded proteins accumulate and become ubiquitinated [17]. They tend to form misfolded protein aggregates and are detected as Ub conjugates. Autophagic adaptor p62-positve puncta colocalized with Ub when PS nanoplastics were allowed to enter the cells (Figure 3B). These results suggest that activated autophagy may recruit ubiquitinated substrates including misfolded protein aggregates for clearance. However, based on the accumulation of Ub conjugates in the presence of PS nanoplastics, it is highly likely that PS nanoplastics may interfere with normal autophagic function or autophagic flux inside the cells.

### 2.3. Clearance of PS Nanoplastics from the Cytoplasm of MEFs

**Figure 4:**
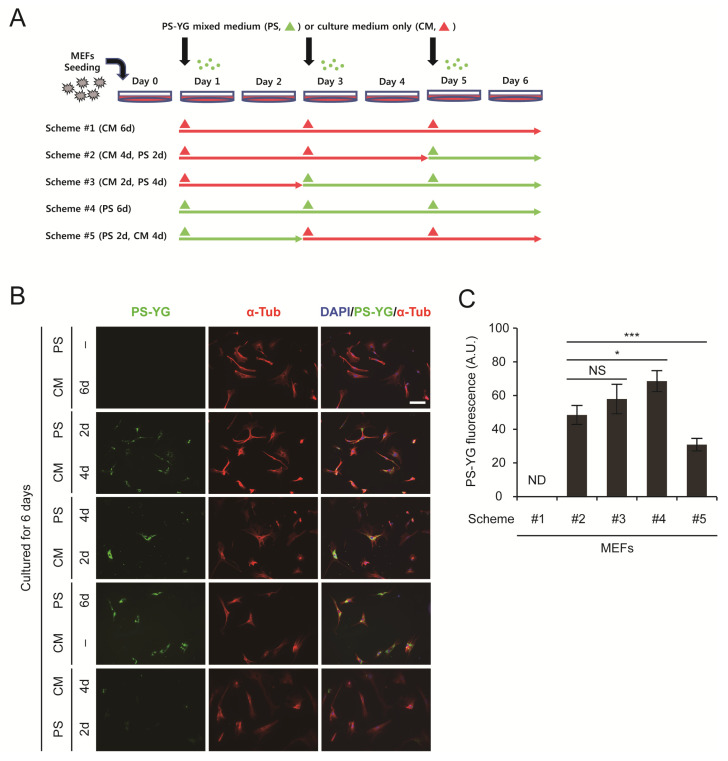
Clearance of PS nanoplastics from the cytoplasm of MEFs. (**A**) Schematic representation of experiments. (**B**) MEFs were treated with 100 nm PS-YG nanoplastics at 200 mg/L for 2 to 6 days. Cells were subjected to immunofluorescence analysis using an anti-α-tubulin (α-Tub) antibody and DNA was visualized with DAPI. Direct fluorescence from PS-YG nanoplastics was also visualized. (**C**) Quantitative analysis of PS-YG fluorescence levels in (B). Green fluorescence levels in all α-Tub-positive cells in the field were determined and expressed as the means ± SEM from the number of cells shown. PS, PS-YG mixed medium. CM, culture medium only. * *p* < 0.05; *** *p* < 0.001 between two groups, as indicated by horizontal bars. ND, not determined. NS, not significant. Scale bar, 100 μm.

Until now, when monitoring the effect of PS nanoplastics on MEFs, we have always cultured cells in medium containing PS nanoplastics. Under these conditions, MEFs were always exposed to PS nanoplastics, and it is not known whether they could be removed from the cells because uptake via endocytosis occurred continuously. To overcome this problem, we decided to wash out PS nanoplastics from the culture medium. In this experiment, we cultured MEFs according to 5 different schemes (Figure 4A). As expected, PS nanoplastics accumulated in the cytoplasm when MEFs were exposed for 2 to 6 days (Figure 4B,C). However, after exposure to PS nanoplastics for 2 days, when PS nanoplastics were removed from the medium and cultured for another 4 days, we found a marked reduction in PS-YG fluorescence inside the cells (Figure 4B, CM 4d, PS 2d vs. PS 2d, CM 4d). These results suggest that PS nanoplastics accumulated in the cytoplasm can be removed or exported out of the cells. Although the exact mechanism for the clearance of PS nanoplastics has not yet been determined, MEFs can tolerate the presence of PS nanoplastics by activating the stress response system and by promoting the clearance of PS nanoplastics from the cytoplasm.

## 3. Discussion

In this study, we demonstrated that PS nanoplastics enter MEFs via endocytosis and affect cellular functions by inducing oxidative and inflammatory stress and interfering with autophagic flux or clearance. Abnormal autophagic function can be caused by the presence of PS nanoplastics in the autophagosome or lysosome.

When MEFs were exposed to PS nanoplastics, it is also possible that PS nanoplastics may adhere to the cell surface without being internalized into the cells. However, we can rule out this possibility because PS nanoplastics were only observed in the cytoplasm—not in the nucleus—of MEFs grown in two dimensions. If PS nanoplastics were attached on the surface of two-dimensional cells, PS nanoplastics could have been observed regardless of the cellular compartments. Moreover, accumulation of PS nanoplastics occurred only in the cytoplasm at juxtanuclear position, but not inside the nucleus (unpublished data). Since the central channel width of the nuclear pore complex (NPC) is less than 50 nm, it is clear that PS nanoplastics with a diameter of 100 nm cannot pass through the NPC. It is intriguing that PS nanoplastics accumulate at juxtanuclear position. While they are in endosomes, the dynein motor protein may be responsible for their retrograde transport along microtubules. As misfolded protein aggregates are also transported in the same manner [18], it would be interesting to further investigate whether PS nanoparticles interact with protein aggregates before being recognized by the autophagic machinery.

It has been thought that PS nanoplastics can induce oxidative stress even when they make contact with the cell surface without internalization. However, our data suggest that the generation of ROS and oxidative stress were most pronounced when PS nanoplastics entered the cells and accumulated in the cytoplasm. Oxidative stress can cause protein misfolding, and when misfolded proteins are not degraded by the proteasome in a timely manner, they tend to from aggregates [17]. These aggregates are ubiquitinated and recognized by the autophagic adaptor p62, and must be cleared through the autophagic pathway [19,20]. However, if PS nanoplastics interfere with the autophagic clearance, these aggregates may accumulate, which are observed as increased levels of Ub conjugates.

According to our data, PS nanoplastics may interfere with the autophagic pathway regardless of whether they remain in endosomes or are released by endosomal escape. If they are in endosomes, they may end up in lysosomes. As PS nanoplastics are resistant to acidic environments, they are not degraded, but PS-containing lysosomes accumulate in the cytoplasm, which may interfere with the autophagic clearance when fused with autophagosomes. On the other hand, if PS nanoplastics are released into the cytoplasm, they may be recognized by the non-selective macroautophagic pathway and incorporated into the autophagosome. This PS-containing autophagosomes may be fused with functional lysosomes, but PS nanoplastics cannot be degraded. Therefore, in both situations, lysosomal dysfunction and impaired autophagic flux could occur.

It is also possible that autophagosomes can be fused with endosomes to form amphisomes, which may contain PS nanoplastics [21,22,23,24]. PS nanoplastics in amphisomes can originate from autophagosomes, endosomes, or both. Therefore, PS nanoplastics either remain in the endosome or are captured by the autophagosome following endosomal escape, and may end up in the amphisome. The amphisome fuses with the lysosome, but PS nanoplastics cannot be degraded in the lysosomal compartment, causing lysosomal dysfunction. PS nanoparticles in lysosomes can be directly exported through lysosome secretion [5]. Alternatively, they can be exported via exosome. For accurate determination how PS nanoplastics are removed or exported out of the cells, further in-depth investigations are required.

Despite oxidative stress and potentially detrimental effects on the autophagic pathway, MEFs were viable and seemed to overcome the effects of PS nanoplastics. We propose that MEFs can reduce the burden by activating the stress response system and removing PS nanoplastics out of the cells.

## 4. Materials and Methods

### 4.1. Isolation and Culture of Mouse Embryonic Fibroblasts

Mouse embryonic fibroblasts (MEFs) were isolated from mouse embryos at 13.5 dpc and cultured as previously described [25]. Briefly, MEFs were cultured in complete medium (Dulbecco’s modified Eagle medium supplemented with 10% fetal bovine serum, 20 mM glutamine, and 1% antibiotics/antimycotics) at 37 °C, with 95% air and 5% CO_2_.

### 4.2. Polystyrene Nanoplastic Treatment

Polystyrene (PS) nanoplastic treatment was carried out as previously described [4]. Non-fluorescent PS or fluorescent PS-YG (yellow-green) nanoplastics with a 100 nm diameter were used in this study (Polysciences, Warrington, PA, USA). According to the manufacturer’s data sheet, PS or PS-YG nanoplastics were provided as a 2.5% (*w*/*v*) aqueous suspension in ddH_2_O or 4.55 × 10^13^ particles/mL with a density of 1.05 g/cm^3^. The properties of PS nanoplastics were characterized in our previous report [4]. Briefly, physical properties, such as size and shape of the PS nanoplastics, were confirmed using a field-emission scanning electron microscope (SU8010; Hitachi, Tokyo, Japan). The dispersion properties, such as hydrodynamic diameter and zeta potential of the PS nanoplastics, were measured using a dynamic light scattering (DLS) instrument (Zetasizer Nano, Malvern Instruments Ltd., Malvern, UK). According to DLS data, PS nanoplastics did not aggregate in solution. For treatment, the PS nanoplastic stock solution was diluted to 200, 500, or 1000 mg/L with complete medium.

### 4.3. Blocking Endocytosis

To block endocytosis, MEFs were treated with sodium azide (NaN_3_) (#S2002, Sigma Aldrich, St. Louis, MO, USA) at 1 day after seeding (DIV1). Sodium azide was diluted to 0.03% (*w*/*v*) in complete medium and treated for 16 h at 37 °C. Sodium azide-containing medium was then removed and PS or PS-YG mixed medium was treated thereafter.

### 4.4. Reactive Oxygen Species Detection Assay

For reactive oxygen species (ROS) detection assay, MEFs were seeded on a 96-well plate. Before the assay, endocytosis was blocked and cells were treated with PS nanoplastics, if necessary. ROS detection assay was performed using an ROS-ID^®^ Total ROS detection kit following the manufacturer’s protocol (#ENZ-51011, Enzo Life Sciences, Farmingdale, NY, USA). Briefly, cells were washed with PBS and ROS detection solution was added (100 μL/well). After 1 h incubation at 37 °C in a dark chamber, the fluorescence was measured using a fluorescence microplate reader (SpectraMax M2^e^, Molecular Devices, San Jose, CA, USA). Fluorescence (RFU, relative fluorescence units) was read from the bottom of the plate at excitation wavelength of 488 nm and emission wavelength of 520 nm. The background fluorescence was subtracted from each RFU.

### 4.5. Quantitative Reverse Transcription PCR

Quantitative reverse transcription PCR (qRT-PCR) was carried out as previously described [4]. Briefly, total RNA was isolated from cultured MEFs using TRI Reagent (Molecular Research Center, Cincinnati, OH, USA), and treated with DNase I (amplification grade; Invitrogen, Carlsbad, CA, USA) for 15 min at room temperature (RT) before reverse transcription. Total RNA (1 μg) was used for reverse transcription using SuperiorScript II Reverse Transcriptase (Enzynomics, Daejeon, Korea), and 1/20 of the synthesized cDNA was used as a template for qRT-PCR. qRT-PCR was performed using a SYBR qPCR 2× Master Mix (Enzynomics) and iCycler system with iCycler iQ software version 2.0 (Bio-Rad, Hercules, CA, USA). The mRNA expression levels of early endosome antigen 1 (*Eea1*), heme oxygenase 1 (*Hmox1*), tumor necrosis factor-α (*Tnfa*), and interleukin-1β (*Il1b*) were normalized to the levels of *Gapdh*. The primers used for qRT-PCR were as follows: *Eea1*-F, 5′-GTG GCA GTC TAG TCA ACG-3′; *Eea1*-R, 5′-CTT CGC CTT TAA GAC ACC TC-3′; *Hmox1*-F, 5′-CCT GGT GCA AGA TAC TGC CC-3′; *Hmox1*-R, 5′-GAA GCT GAG AGT GAG GAC CCA-3′; *Tnfa*-F, 5′-TCT CAT CAG TTC TAT GGC CC-3′; *Tnfa*-R, 5′-GGG AGT AGA CAA GGT ACA AC-3′; *Il1b*-F, 5′-TTG ACG GAC CCC AAA AGA TG-3′; *Il1b*-R, 5′-AGA AGG TGC TCA TGT CCT CA-3′; *Gapdh*-F, 5′-GGC ATT GCT CTC AAT GAC AA-3′; and *Gapdh*-R, 5′ -CTT GCT CAG TGT CCT TGC TG-3′.

### 4.6. Immunoblot Analysis

Immunoblot analysis was performed as previously described [4]. Briefly, cell lysates were prepared in lysis buffer (50 mM Tris-HCl [pH 7.5], 200 mM NaCl, 1% NP-40, 1% sodium deoxycholate) with 1 mM PMSF, 1 μg/μL aprotinin, and 1 μg/μL leupeptin as protease inhibitors and incubated on ice for 30 min. Total cell lysates (10 μg) were subjected to SDS-PAGE, followed by immunoblot detection with anti-Eea1 (1:1000, #MA5-14794, Invitrogen), anti-Hmox1 (1:1000, #5061, Cell Signaling Technology, Danvers, MA, USA), anti-Nqo1 (1:1000, #ab34173, Abcam, Cambridge, UK), anti-LC3B (1:1000, #2775, Cell Signaling Technology), anti-Ub(P4D1) (1:500, #8017, Santa Cruz Biotechnology, Dallas, TX, USA), anti-α-tubulin (1:500, #32293, Santa Cruz Biotechnology), or anti-β-actin antibodies (1:500, Santa Cruz Biotechnology). Based on the type of primary antibody, the appropriate HRP-conjugated goat anti-mouse or anti-rabbit IgG (1:10,000; Enzo Life Sciences) was used.

### 4.7. Immunofluorescence Analysis

Immunofluorescence analysis was performed as previously described [4]. Briefly, MEFs were grown on poly-D-lysine-coated coverslips and were fixed in 4% paraformaldehyde for 10 min at RT, permeabilized with 0.3% Triton X-100/PBS for 5 min at RT, blocked with 3% BSA/PBS for 1 h at RT, and incubated with anti-α-tubulin (1:250, #32293, Santa Cruz Biotechnology), anti-LC3B (1:500, #2775, Cell Signaling Technology), anti-SQSTM1/p62 (1:500, #5114, Cell Signaling Technology), or anti-Ub(FK2) (1:1000, #04-263, Millipore, Burlington, MA, USA) antibodies at 4 °C overnight. They were then incubated with Alexa Fluor 555- or 633-conjugated goat anti-mouse or donkey anti-rabbit IgG (1:1000, Invitrogen) with 0.1 μg/mL 4′,6-diamidino-2-phenylindole (DAPI) for 1 h at RT. Cells were then mounted onto slides using Prolong Gold antifade reagent (Invitrogen). Immunofluorescence images were visualized with an Axio Imager A2 microscope or an Axio Observer 7 microscope equipped with an LSM 800 confocal laser-scanning module (Carl Zeiss, Oberkochen, Germany). If needed, PS-YG fluorescence levels in cells were quantified using an ImageJ ROI (region of interest) manager software (1.53e).

### 4.8. Statistical Analysis

Two-tailed unpaired Student’s *t*-test or two-way analysis of variance (ANOVA) followed by post hoc Tukey’s test were used to compare data between two groups. *p* < 0.05 was considered to be statistically significant.

## Data Availability

Not applicable.
